# Copper(II) invigorated EHU-30 for continuous electroreduction of CO_2_ into value-added chemicals

**DOI:** 10.1038/s41598-022-11846-w

**Published:** 2022-05-20

**Authors:** Nerea Landaluce, Maite Perfecto-Irigaray, Jonathan Albo, Garikoitz Beobide, Oscar Castillo, Angel Irabien, Antonio Luque, Alba S. J. Méndez, Ana E. Platero-Prats, Sonia Pérez-Yáñez

**Affiliations:** 1 Department of Organic and Inorganic Chemistry, University of the Basque Country, UPV/EHU, P.O. 644, 48080 Bilbao, Spain; 2 Departamento de Ingenierías Química y Biomolecular, Universidad de Cantabria, 39005 Santander, Spain; 3 BCMaterials, Basque Center for Materials, Applications and Nanostructures, UPV/EHU Science Park, 48940 Leioa, Spain; 4 Bayerisches Geoinstitut, University of Bayreuth, Bayreuth, Germany; 5 Departamento de Química Inorgánica, Universidad Autónoma de Madrid, Madrid, Spain; 6 Condensed Matter Physics Center (IFIMAC), Madrid, Spain; 7 Institute for Advanced Research in Chemical Sciences (IAdChem), Universidad Autónoma de Madrid, Madrid, Spain

**Keywords:** Inorganic chemistry, Metal-organic frameworks

## Abstract

The doping of zirconium based EHU-30 and EHU-30-NH_2_ metal–organic frameworks with copper(II) yielded a homogeneous distribution of the dopant with a copper/zirconium ratio of 0.04–0.05. The doping mechanism is analysed by chemical analysis, microstructural analysis and pair distribution function (PDF) analysis of synchrotron total scattering data in order to get deeper insight into the local structure. According to these data, the Cu(II) atoms are assembled within the secondary building unit by a transmetalation reaction, contrarily to UiO-66 series in which the post-synthetic metalation of the MOF takes place through chemical anchorage. The resulting materials doubled the overall performance of the parent compounds for the CO_2_ electroreduction, while retained stable the performance during continuous transformation reaction.

## Introduction

CO_2_ emissions are a major environmental concern which can be mitigated in the near future if efficient capture, storage and revalorization solutions are developed^1,2^. In this context, the conversion of CO_2_ into useful chemicals and fuels implies an appealing solution that could potentially pave the transition to a low-carbon economy^3,4^. The conversion process can be conducted by thermochemical, photochemical, biochemical and electrochemical methods^5,6^. In particular, electroreduction of CO_2_ has received a great deal of attention, due to its simple procedure and smooth reaction conditions. Note that the energy required for the electrochemical activation of CO_2_ can be supplied by renewable energy sources, such as solar or wind power, providing a viable solution to yield carbon–neutral fuels and chemicals. Previous researches have concluded that copper based materials are the most suitable electrocatalysts to break C–O bond of CO_2_ and further convert CO to more reduced species such as alcohols and hydrocarbons^7^. Metal–organic frameworks (MOFs) have been posited as promising alternative to conventional copper metal or copper oxide based catalysts, owing to their tuneable chemistry and electronic properties, but also because of their high surface area and porosity that can ease the mass transport limitations^8^. However, despite copper-based MOFs have proven to yield products as ethanol and methanol, they lack of stability upon long-term runs which hinder their application^9,10^. This drawback can be overcome using zirconium-MOFs as electrocatalytic materials, in which the strength of Zr–O provides highly stable coordination network capable of withstand demanding reaction conditions^11^. Nonetheless, when using Zr-based metal–organic materials the reaction progress is limited to the formation of less reduced products such as formic acid^12,13^.

At this point, it deserves to note that the methods to post-synthetically modify MOFs can provide a tailor-made solution when all desired properties and/or functionalities cannot be achieved in the pristine MOF structure. In this regard, MOFs based on zirconium clusters have proved to be suitable platforms to be doped with other transition metal ions, particularly when the linker vacancies around the clusters enables the metalation of the framework by chemical anchoring or transmetalation reactions^14,15^.

## Results and discussion

**Figure 1:**
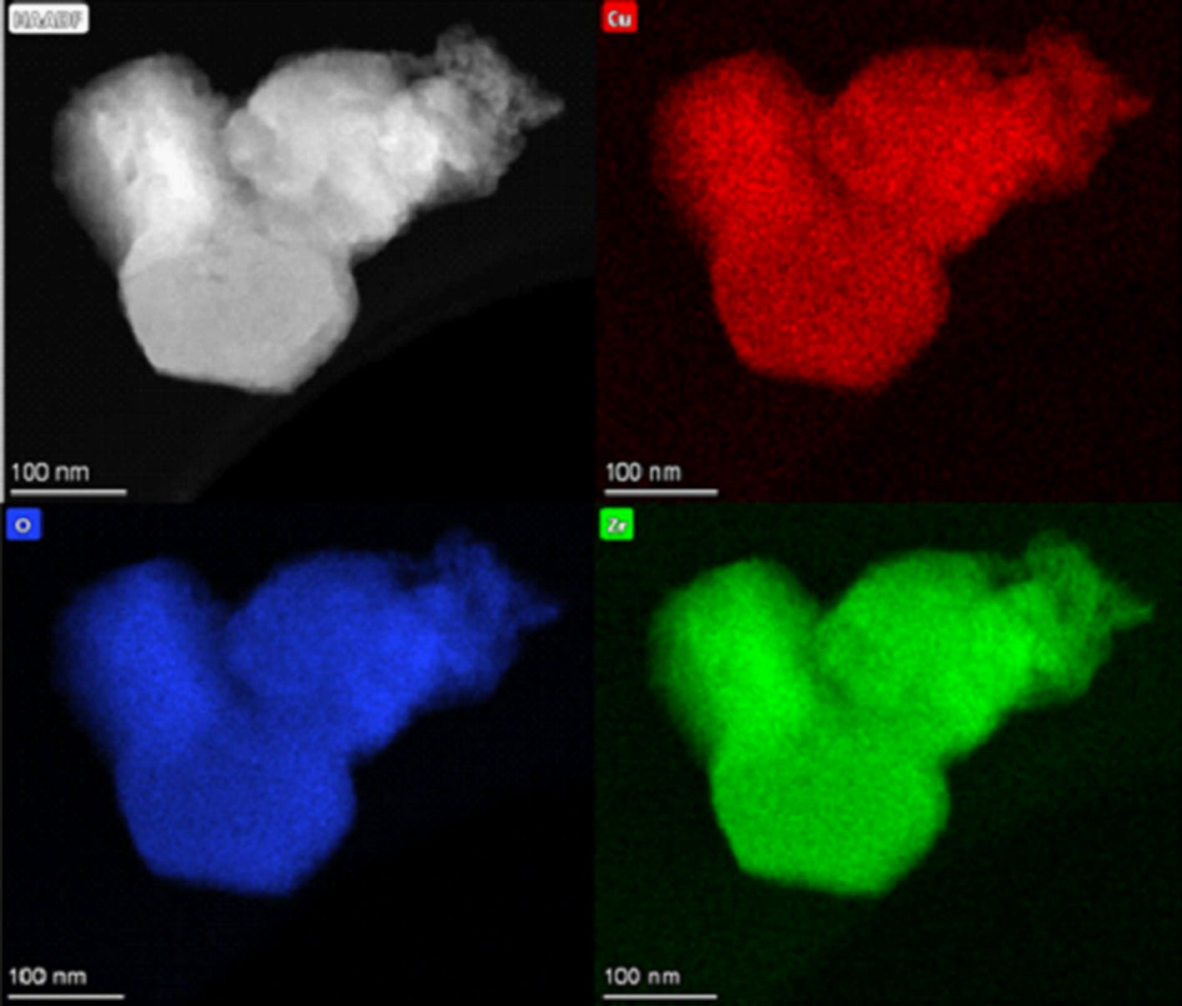
TEM images taken on Cu@EHU-30 showing the elemental distribution determined by EDXRF.

**Figure 2:**
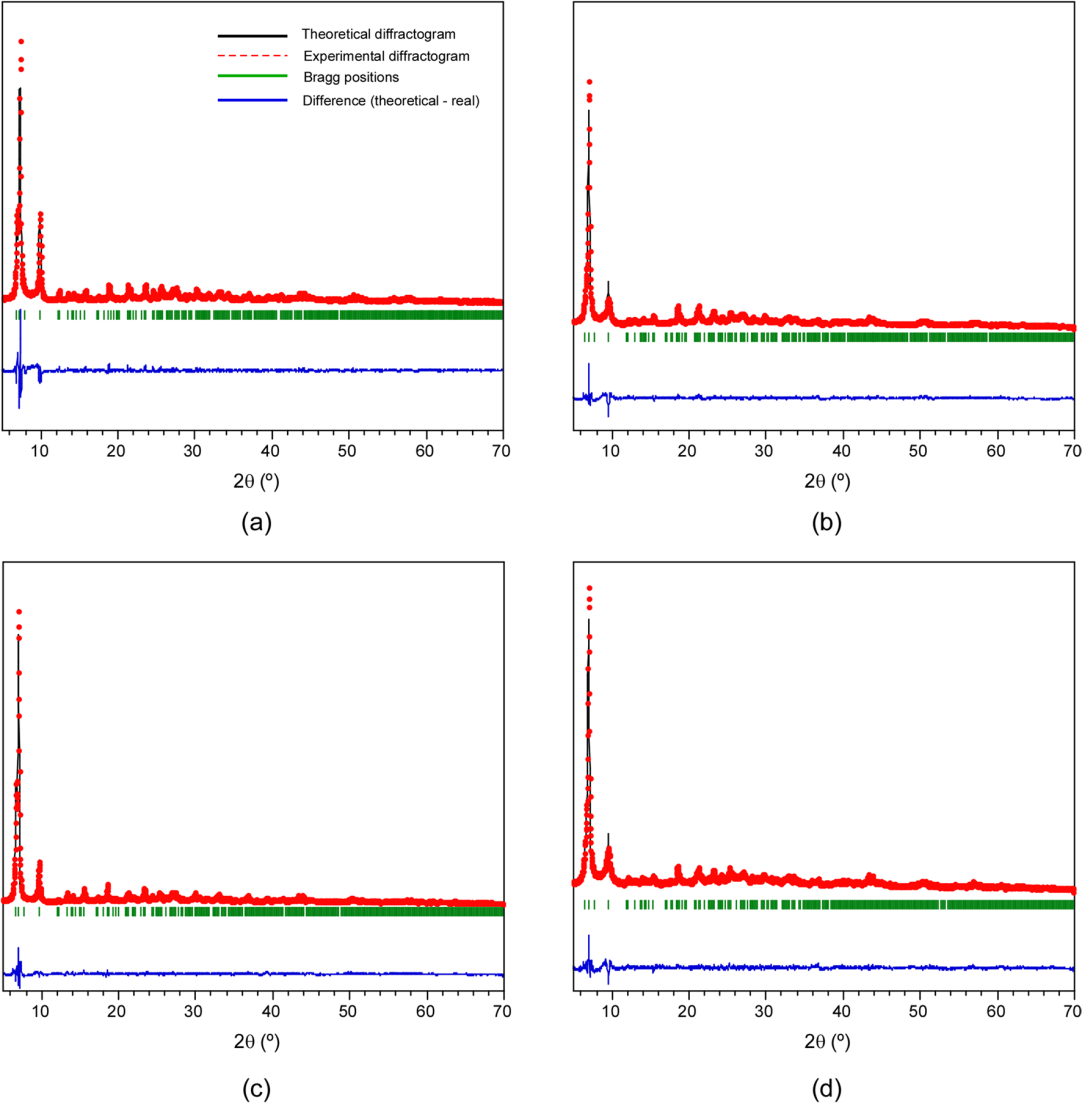
Pattern-matching analysis for (**a**) EHU-30, (**b**) EHU-30-NH_2_, (**c**) Cu@EHU-30 and (**d**) Cu@EHU-30-NH_2_.

In this work, firstly we have synthesised two zirconium MOFs, named EHU-30 and EHU-30-NH_2_, and doped them with copper(II) to analyse their performance in the electroreduction of CO_2_. The structure of these isoreticular MOFs is built by 8-connected [Zr_6_(μ_3_-O)_4_(μ_3_-OH)_4_(μ-COO)_12_] secondary building units (SBUs), linked by twelve linkers (phenyl and aminophenyl rings for EHU-30 and EHU-30-NH_2_, respectively) into a three-dimensional framework with hexagonal primitive topology (**hex**)^16,17^. Both can be regarded as polymorphs of UiO-66 series (12-connected SBUs into a **fcu**-type net) and exhibit analogous bidimensional hexagonal subnets (**hxl**) in which each Zr_6_-cluster is 6-connected by bridging ligands (BDC: benzene-1,4-dicarboxylato; NH_2_BDC: 2-aminobenzene-1,4-dicarboxylato). However, the connection among the 2D subnets in EHU-30 series occurs through a triple bunch of linkers, which are rather stressed and favour the occurrence of structural defects. In fact, the chemical analysis of the herein prepared materials has revealed a 20 and 10% of linker vacancies for EHU-30 and EHU-30-NH_2_, respectively, which are compensated by the presence of modulator molecules and H_2_O/OH^–^ pairs to counterbalance the coordination position and charge of the hexanuclear zirconium cluster (see section S1 of the Supplementary Information). Both MOFs were post-synthetically modified by the reaction with a methanol solution of copper(II) nitrate. As a result, a Cu/Zr ratio of 0.04 and 0.05 was achieved for each MOF (Cu@EHU-30 and Cu@EHU-30-NH_2_), while no traces of nitrate remained in the doped frameworks. Transmission electron microscopy (TEM) images show a homogeneous distribution of the dopant metal over the hexagonal prism shaped crystallites of the MOFs (Fig. 1), while the comparison of powder X-ray diffraction (PXRD) patterns with those of pristine MOFs ensures the phase purity of the samples after doping process (Fig. S2.1). PXRD patterns of all compounds were fitted on the basis of the space group (*P*6_3_/*mmc*) and cell parameters reported for the crystal structures of EHU-30 and EHU-30-NH_2_^16,17^. The fitting parameters and figures of merit are gathered in Tables S2.1–S2.4. According to the profile fitting analysis performed on the PXRD data (Fig. 2), the cell parameters of the parent compounds (EHU-30: a = 14.666(3) Å, b = 26.883(8) Å; EHU-30-NH_2_: a = 14.6741(4) Å, b = 27.363(1) Å) remained almost invariable (shifts: < 1%) after the metalation reaction (Cu@EHU-30: a = 14.6630(2) Å, b = 26.6305(6) Å; EHU-30-NH_2_: a = 14.6955(3) Å, b = 27.305(1) Å). Thus Cu-doping did not exert any meaningful change in the overall crystal structure which is consistent with robustness of the zirconium coordination framework.

**Figure 3:**
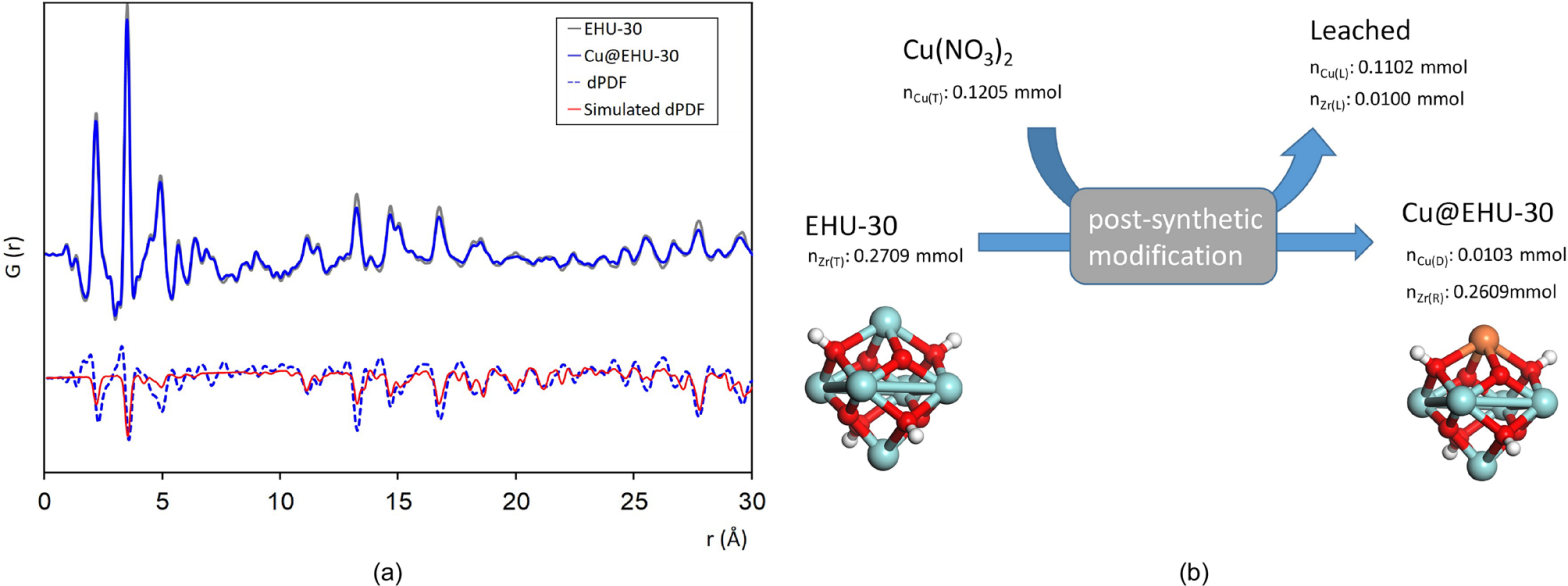
(**a**) Total PDF data of EHU-30 and Cu@EHU-30, and differential PDF signal to highlight changes in intensity. (**b**) Molar balance resulting from the post-synthetic metalation of EHU-30, depicting a representative image of the transmetalation within the inorganic core {M_6_O_4_(OH)_4_} (T: total amount; L: leached; D: doping; R: remanent).

Pair distribution function analysis (PDF) of synchrotron total scattering data of EHU-30 and Cu@EHU-30 (Fig. 3a) show almost coincident profiles, with some subtle differences in the intensity of the signal after normalisation to chemical composition. The simulated PDF data show a decay of the intensity for the atom–atom correlations corresponding to Zr···Zr, Zr–O and Zr···O distances, which suggests that the inclusion of copper takes place through a transmetalation reaction. The absence of new peaks in the PDF profile of Cu@EHU-30 compared to that of the pristine material allows to disregard a chemical anchoring to the cluster or nucleation of copper in the pore cavity. Furthermore, the chemical balance analysis (Fig. 3b) fits the expectations for a transmetalation reaction in which each incorporated copper atom replaces a zirconium atom of the hexanuclear cluster. Note that the high ratio of linker vacancies in EHU-30 series and the stress accumulated on the triple bunch of linkers can facilitate the replacement of Zr from the SBU. In fact, upon similar doping conditions, the metalation with Cu(II) of the polymorphic UiO-66 series takes place through chemical anchorage at the SBU^14^, which seems to support the relevance of the unique structural features of EHU-30 series into the transmetalation reaction. We also observed that upon same doping conditions other divalent first row transition metal ions (Mn, Fe, Co, Ni, Zn) led to markedly smaller metalation (M/Zr ratio: 0.006–0.014, Fig. S1.1) than that achieved for copper, which can be related to the plasticity of the coordination sphere of Cu(II) that can ease its inclusion.

Prior to the electrochemical transformation experiments, the porosity of the pristine and doped samples was assessed by the measurement of dinitrogen and carbon dioxide adsorption isotherms (see Supplementary Information). All the N_2_ (77 K) adsorption curves resemble a type I isotherm with a sharp knee at low relative pressures (P/P_0_ < 0.04), as expected for microporous samples. The slight progressive increase at intermediate pressures and the condensation at relative high pressures (P/P_0_ > 0.94) is explained by the interparticle porosity of the nanosized crystallites. The estimated BET surface area was smaller for doped samples (Cu@EHU-30 and Cu@EHU-30-NH_2_: 801 and 558 m^2^ g^–1^) than for parent compounds (EHU-30 and EHU-30-NH_2_: 1065 and 885 m^2^ g^–1^), which probably could be related to a slight crystallinity decrease led by the post-synthetic modification, as inferred from the increase of full width at half maximum (FWHM) values of the PXRD reflections (13 and 21% increase of FWHM for Cu@EHU-30 and Cu@EHU-NH_2_, respectively). The isosteric heats of adsorption (Q_st_) derived from CO_2_ isotherms show values ranging from 27 to 31 kJ·mol^–1^ at near-zero coverage, which decay to 23–26 kJ mol^–1^ when the gas uptake reaches a ratio of 2.5 CO_2_ per metal cluster. At low coverage, the Q_st_ values of EHU-30-NH_2_ (29.2 kJ mol^–1^) overpasses those provided by EHU-30 (27.8 kJ mol^–1^), which can be explained by the enhanced interacting capacity provided by the amino-group upon the CO_2_ adsorption^18^. Besides, the inclusion of Cu(II) seems to improve also the affinity towards CO_2_ as it also yields somewhat higher Q_st_ values (Cu@EHU-30 and Cu@EHU-30-NH_2_: 28.9 and 30.9 kJ mol^–1^) than those exhibited by the parent compounds.

**Figure 4:**
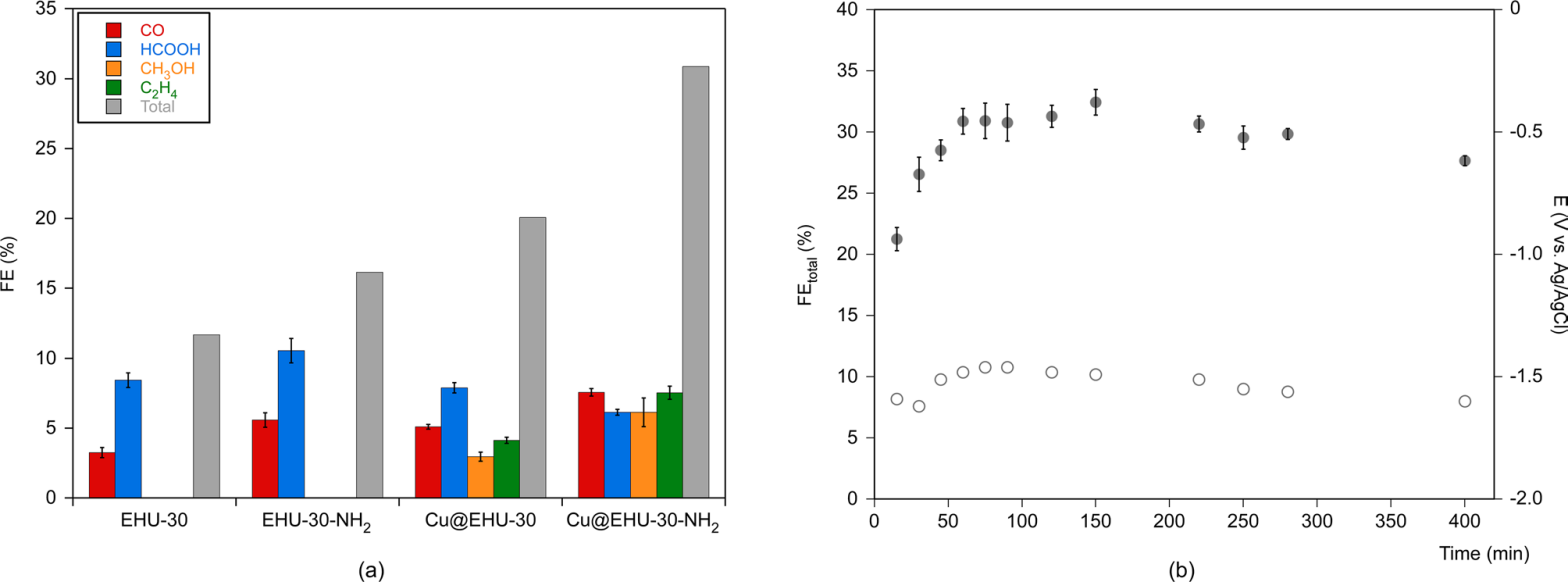
(**a**) FE as a function of the electrocatalytic material applied. (**b**) Time-dependence of total FE (filled circles) and current density/potential (empty circles) for all CO_2_ reduction products using Cu@EHU-30-NH_2_.

Figure 4 gathers the data for the continuous transformation of CO_2_ at galvanostatic conditions in a filter press electrochemical cell in which the pristine and Cu-doped MOFs have been supported in gas diffusion electrodes (GDE) to act as electrocatalyst (see details in SI). The analysis is performed in terms of reaction rates for CO, HCOOH, CH_3_OH and C_2_H_4_ formation, as well as the total rate and cell potential (E). The corresponding Faradaic efficiency (FE) values were calculated considering that 2 e^-^ are required per molecule of CO and HCOOH, 6 e^-^ for CH_3_OH and 12 e^-^ for C_2_H_4_ (Fig. 4a). No other liquid products were detected, except traces of ethanol. H_2_ production (see Table S8.1 of the supplementary information) and energy losses in the cell (ohmic resistances) explained the remaining efficiency percentage. Control experiments for neat carbon papers did not produce any measurable product, so the performance can be ascribed to the catalytic activity of the selected materials.

Neat zirconium MOFs exhibit moderate reaction rates and efficiencies for the CO_2_ conversion into CO and HCOOH. The FE_HCOOH_ values are higher (8.4 and 10.5% for EHU-30 and EHU-30-NH_2_, respectively) than those achieved with other zirconium metal–organic materials (2.3–4.2%) in equal operating conditions^12^. Besides, the amino-function in EHU-30-NH_2_ boosts the yield toward both CO and HCOOH, reaching higher total reaction rates and FE values than the not functionalised EHU-30 (61.9 vs 47.9 µmol m^-2^ s^-1^; 16.1 vs 11.7%). This improvement might be related with the greater affinity towards CO_2_ uptake provided by the amino-functionalization, as demonstrated by the measured isosteric heats of adsorption. In fact, previous works have stated that the inclusion of amino functional groups favours CO_2_ adsorption and, as a consequence, CO_2_ reduction reaction is enhanced with respect to the HER^19^. This trend is also observed in the series of MOF herein analysed (see Table S8.1). Note that, as described below, the inclusion of copper(II) seems to have the same effect and therefore, the overall performance could be ascribed to a dual active site system.

As it was aimed, the inclusion of copper(II) promotes a further conversion of CO_2_ into more reduced species, i.e. methanol and ethylene, while the CO and HCOOH yields are almost retained. As a result, the total reaction rates and Faradaic efficiency experience a meaningful enhancement doubling the performance of pristine MOFs (Cu@EHU-30: 74.0 µmol m^-2^ s^-1^ and 20.1%; Cu@EHU-30-NH_2_: 109.3 µmol m^-2^ s^-1^ and 30.9%). Considering the previously reported results for homometallic Cu^II^-MOFs and heterometallic Cu^II^/M-MOFs (M: Zn^II^, Ru^III^, Pd^II^)^9,19^, the herein prepared materials provide a similar yield of the most reduced products (alcohols and alkenes) and a greater performance when considering all products, despite of the relative low amount of copper(II) used in the doped samples (Cu@EHU-30 and Cu@EHU-30-NH_2_: 9.1 and 11 mg Cu/g MOF; reported copper-based MOFs: 123–350 mg Cu/g MOF). Such behaviour can be ascribed to a synergic relation implying both zirconium and copper metal centres. In this sense, formic acid can be considered an intermediate product in the formation of alcohols and alkenes. Thus, its concentration in the reaction media limits the yield toward the more reduced species that Cu containing MOFs can yield. Since Cu^II^-MOFs lead to rather low formation of formic acid and zirconium based materials yields formic acid as the mayor product, the simultaneous presence of both metal centres invigorates the overall performance of the reaction. Similar results were observed for the physical mixture of polycrystalline MOFs of Cu(II) and Bi(III), but using greater amounts of copper (52–289 mg Cu/g MOF)^10^.

To assess the long term stability of the neat and Cu-doped zirconium MOFs, the continuous transformation of CO_2_ was monitored for 400 min. The resulting time dependence for the total FE for Cu@EHU-30-NH_2_ is plotted in Fig. 4b along with the current/density potential over time. The results show a slight increase of the performance during the first 90 min to get into a roughly steady conversion of CO_2_. This stable conversion is related to the stability provided by the zirconium-based coordination framework. Note that despite some material leaching occurs during the reaction, the microstructural and chemical analysis (see details in the SI) indicate that the overall structure of the coordination framework is retained, including the amino group of the ligand. Such performance stability implies a considerable advantage with respect previously reported Cu-based MOF^9,20^ for which the performance decays at reaction times shorter than 1 h. Consider that Cu-based MOFs imply a framework built by the assembly of Cu-SBUs and carboxylate linkers, which provide a feebler structure than that based on zirconium-carboxylate bonding^21^.

On the other hand, the slight initial increase in the FE values (Fig. 4b) could be related to the anchoring to the MOF structure of the initially produced formic acid which is also a likely intermediate in the formation of more reduced species. Its conjugate base, formate anion, is a ligand that shows a noteworthy trend to coordinate to Zr(IV) and in fact, is found in many Zr-MOF replacing OH^–^/H_2_O pairs, as it occurs in MOF-808, MOF-802 and DUT-67, among others^22^. Thus a plausible explanation might be provided by the fact that part of the formic acid formed in the initial stage is coordinated to the linker vacancies of the MOF replacing OH^–^/H_2_O (i.e. some of the initially formed formic acid is trapped in the solid). Once the linker vacancies are saturated the measured FE can rise to reach roughly stable maximum value. In fact, a similar trend in FE values was observed in a previous work in which a family of Zr based metal–organic gels was reported. These gels presented many ligand vacancies and they showed a similar trend for FE values corresponding to formic acid production^12^.

## Conclusions

EHU-30 and EHU-30-NH_2_ zirconium-based MOFs were doped with Cu(II) at Cu/Zr ratios of 0.04 and 0.05, displaying a homogeneous distribution of copper over the MOF nanoscopic crystallites. The chemical balance and PDF analysis suggest a transmetalation reaction in such a way that each incorporated copper atom replaces a zirconium atom of the metal cluster that comprises the SBU of the MOF. When comparing the results of the CO_2_ electroreduction reactions, the MOFs containing the amino functional group (EHU-30-NH_2_ and Cu@EHU-30-NH_2_) led to a better performance than their not functionalised analogous materials (EHU-30 and Cu@EHU-30), probably, due to the favoured CO_2_ adsorption of the formers. Besides, while neat zirconium MOFs produce CO and HCOOH as the major products, doping them with copper turns the product selectivity toward more reduced species (methanol and ethylene) but also enhances markedly the overall performance doubling the total reaction rates and Faradaic efficiencies of neat zirconium MOFs. Apart from that, the inclusion of copper as dopant into a zirconium metal–organic framework provides a roughly stable continuous transformation of CO_2_ into value-added products and thus, it precludes the rapid deactivation reported for Cu-based MOFs, which is a major obstacle for the application of this kind of materials in CO_2_ electroreduction reactions.

## Methods

### Synthesis and doping of EHU-30 and EHU-30-NH_2_

EHU-30 and EHU-30-NH_2_ were prepared following procedures similar to previously reported ones^16,17^. See details below. Chemical characterization of the samples was completed by PXRD, FTIR, TGA, H-NMR and ICP-OES (see below).

#### Synthesis of EHU‐30

Zr(OPr)_4_ (1.050 g, 2.25 mmol) and benzene-1,4-dicarboxylic acid (0.4 g, 2.4 mmol) were added into a sealed glass reactor. The mixture was stirred for 5 min, and then methacrylic acid was added (700 μL). The mixture was stirred for 10 min. Lastly, distilled water (10 μL) was added and the doughy mixture was stirred for 30 min. Thereafter, the flask was sealed again and the mixture was heated to 140 °C for 2 h under continuous stirring. To the obtained product, MeOH (20 mL) was added and the mixture was stirred for 2 h. The obtained product was washed three times with methanol, filtered under vacuum and dried at 130 °C. The resulting compound presented the aspect of a finely divided white powder.

#### Synthesis of EHU‐30‐NH_2_

Zirconium(IV) propoxide (1.053 g, 2.25 mmol), isobutyric acid (1400 µL, 15 mmol) and 2‐aminobenzene‐1,4‐dicarboxylic acid (0.412 g, 2.25 mmol) were mixed under continuous stirring in a teflon vessel. Thereafter, 10 µL (0.56 mmol) of water were added. The closed teflon vessel was placed in a preheated oven at 140 °C for 4 h. Again, the synthesis product was washed three times with methanol, filtered under vacuum and dried at 130 °C. The resulting compound appeared as yellowish fine powder.

#### Synthesis of Cu@EHU‐30 and Cu@EHU-30-NH_2_

MeOH (7 mL) was added to the copper(II) nitrate (0.017 g, 0.072 mmol), and it was stirred until it became a clear solution. Then, EHU-30 (0.12 g, 0.072 mmol) was added and the suspension was stirred at room temperature for 2 h. Consecutively, the powdered sample was collected by vacuum filtration and washed with MeOH (7 mL) for 10 min to remove unreacted copper(II) salt. Finally, the obtained precipitate was collected again by vacuum filtration and dried at 80 °C for 1 h. For comparative purposes the doping process was also performed for other metal transition metal ions following the same procedure but using instead copper(II) nitrate, manganese(II) nitrate, iron(II) chloride, cobalt(II) nitrate, nickel(II) nitrate hexahydrate or zinc nitrate hexahydrate.

### Physical measurements

X-ray diffraction data was obtained using a PANalytical Xpert PRO difractometer (equipped with Cu-K_α_ radiation, λ = 1.5418 Å) over the range 5 < 2θ < 70°, programmable divergence slit, automatic sample exchanger and PixCel detector. The thermal stability of the compounds was studied by thermogravimetry in a TGA / SSDTA 851 Mettler Toledo unit under a synthetic air atmosphere (80% N_2_ and 20% O_2_), from room temperature up to 800 °C, with a heating ramp of 5 °C / min. The infrared spectra were carried out with the FTIR 8400S Shimadzu equipment in the interval of 500 to 4000 cm^–1^, employing the ATR module. ^1^H-RMN spectrum was acquired in a Bruker AVANCE 500 (one-bay; 500 MHz) at 293 K. The sample (0.7 g) was dissolved in a mixture of 2 mL of D_2_O with 0.8 g of NaOH while stirring for 24 h. X-ray fluorescence (XRF) data was acquired by MIDEX SD micro fluorescence X-ray spectrometer (Spectro) using ED-XRF energy dispersion for elemental analysis. It has an automatic XYZ tray, an automatic collimator changer, an X-ray tube with Mo anode and a silicon drift detector (SDD) with an area of 30 mm^2^.

Inductively coupled plasma optical emission spectrometry (ICP-OES) data was obtained by Agilent 5100 with dual vision, axial and radial. Plasma flow was 12 L·min^–1^, nebulization of 0.7 mL·min^-1^ and auxiliary 1.0 mL·min^–1^ with 1300 W of power. The samples were dissolved with 35 mL of HNO_3_ and 15 mL of HCl in a microwave equipment at 200 °C. Elemental analysis of carbon, hydrogen and nitrogen (CHN analysis) data was achieved by Eurovector EA 3000. Calibration samples were prepared in tin capsules packed with acetanilide. The prepared calibration and analysis samples were placed in the auto-sampler from where they were periodically tipped into a vertical quartz reactor heated at a temperature of 980 °C with a constant flow of helium stream. The resulting components N_2_, CO_2_, H_2_O are separated in a chromatographic column and detected by a thermo-conductivity detector. The obtained signals were analyzed by Callidus® software.

To obtain physisorption data, the Autosorb iQ Quantachrome Instruments analyzer was used. Prior to measurement all the samples were outgassed at 140 °C during 6 h. The N_2_ adsorption isotherms of the compounds were measured at 77 K and the CO_2_ adsorption isotherms were measured at 273 and 298 K.

Transmission electron microscopy (TEM) was performed on a TECNAI G2 20 TWIN operated at 200 kV and equipped with LaB_6_ filament and an energy-dispersive X-ray (EDX) spectrometer. The samples for the TEM were prepared by dispersion into ethanol solvent and keeping the suspension in an ultrasonic bath for 15 min. Thereafter a drop of the suspension was spread onto a TEM copper grid (300 Mesh) covered by a holey carbon film followed by drying under vacuum.

## Supplementary Information

Supplementary Information.

## Data Availability

The datasets used and/or analysed during the current study available from the corresponding author on reasonable request.
