# Supplementary material for: Copper(II) invigorated EHU-30 for continuous electroreduction of CO2 into value-added chemicals
Source: Sci Rep. 2022 May 20;12:8505. doi: 10.1038/s41598-022-11846-w (PMC9123010; doi:10.1038/s41598-022-11846-w)
Supplement: Supplementary file 1 — Supplementary Information. [file 41598_2022_11846_MOESM1_ESM.pdf]

Supplementary Information for

Copper(II) invigorated EHU-30 for continuous electroreduction of CO_2 into value-added chemicals

Nerea Landaluce,[1] Maite Perfecto-Irigaray,[1] Jonathan Albo,[2] Garikoitz Beobide,*[1,3] Oscar Castillo,*[1,3] Angel Irabien,[2] Antonio Luque,[1,3] Alba S. J. Méndez,[4] Ana E. Platero-Prats[5,6] and Sonia Pérez-Yáñez[1,3]

[1]Department of Organic and Inorganic Chemistry, University of the Basque Country, UPV/EHU, P.O. 644, E-48080 Bilbao, Spain.
[2]Departamento de Ingenierías Química y Biomolecular, Universidad de Cantabria, E-39005 Santander, Spain
[3]BCMaterials, Basque Center for Materials, Applications and Nanostructures, UPV/EHU Science Park, E-48940 Leioa, Spain.
[4]Bayerisches Geoinstitut, University of Bayreuth, Bayreuth, Germany
[5]Departamento de Química Inorgánica, Universidad Autónoma de Madrid, Madrid, Spain
[6]Condensed Matter Physics Center (IFIMAC), Madrid, Spain
* garikoitz.beobide@ehu.eus, oscar.castillo@ehu.eus

S0. PHYSICAL MEASUREMENTS 2

S1. SYNTHESIS AND DOPING OF EHU-30 AND EHU-30-NH_2 4

S2. POWDER X-RAY DIFFRACTION (PXRD) ANALYSIS 7

S3. FOURIER-TRANSFORM INFRARED SPECTRA (FTIR) 10

S4. NUCLEAR MAGNETIC RESONANCE (NMR) SPECTROSCOPY 12

S5. THERMOGRAVIMETRIC ANALYSIS 15

S6. SYNCHROTRON TOTAL SCATTERING DATA AND PAIR DISTRIBUTION FUNCTION ANALYSIS 17

S7. GAS ADSORPTION DATA 19

S8. CONTINUOUS CO_2 ELECTROREDUCTION EXPERIMENTS 23

S9. POST-REACTION CHARACTERIZATION 26

S10. REFERENCES 26

S0. PHYSICAL MEASUREMENTS

S0. PHYSICAL MEASUREMENTS

X-ray diffraction data was obtained using a PANalytical Xpert PRO difractometer (equipped with Cu-K_α radiation, λ=1.5418 Å) over the range 5<2θ<70°, programmable divergence slit, automatic sample exchanger and PixCel detector. The thermal stability of the compounds was studied by thermogravimetry in a TGA / SSDTA 851 Mettler Toledo unit under a synthetic air atmosphere (80% N_2 and 20% O_2), from room temperature up to 800 °C, with a heating ramp of 5 °C / min. The infrared spectra were carried out with the FTIR 8400S Shimadzu equipment in the interval of 500 to 4000 cm^{-1}, employing the ATR module. ^{1}H-NMR spectrum was acquired in a Bruker AVANCE 500 (one-bay; 500 MHz) at 293 K. The sample (0.7 g) was dissolved in a mixture of 2 mL of D_2O with 0.8 g of NaOH while stirring for 24h. X-ray fluorescence (XRF) data was acquired by MIDEX SD micro fluorescence X-ray spectrometer (Spectro) using ED-XRF energy dispersion for elemental analysis. It has an automatic XYZ tray, an automatic collimator changer, an X-ray tube with Mo anode and a silicon drift detector (SDD) with an area of 30 mm^2.

Inductively coupled plasma optical emission spectrometry (ICP-OES) data was obtained by Agilent 5100 with dual vision, axial and radial. Plasma flow was 12 L·min^{-1}, nebulization of 0.7 mL·min^{-1} and auxiliary 1.0 mL·min^{-1} with 1300W of power. The samples (70 mg) were dissolved with 35 mL of HNO_3 and 15 mL of HCl in a microwave equipment at 200 °C. Elemental analysis of carbon, hydrogen and nitrogen (CHN analysis) data was achieved by Eurovector EA 3000. Calibration samples were prepared in tin capsules packed with acetanilide. The prepared calibration and analysis samples were placed in the auto-sampler from where they were periodically tipped into a vertical quartz reactor heated at a temperature of 980 °C with a constant flow of helium stream. The resulting components N_2, CO_2, H_2O are separated in a chromatographic column and detected by a thermo-conductivity detector. The obtained signals were analyzed by Callidus® software.

To obtain physisorption data, the Autosorb iQ Quantachrome Instruments analyzer was used. Prior to measurement all the samples were outgassed at 140 °C during 6 h. The N_2 adsorption isotherms of the compounds were measured at 77 K and the CO_2 adsorption isotherms were measured at 273 and 298 K.

Transmission electron microscopy (TEM) was performed on a TECNAI G2 20 TWIN operated at 200 kV and equipped with LaB_6 filament and an energy-dispersive X-ray (EDX) spectrometer. The samples for the TEM were prepared by dispersion into ethanol solvent and keeping the suspension in an ultrasonic bath for 15 min. Thereafter a drop of the suspension was spread onto a TEM copper grid (300 Mesh) covered by a holey carbon film followed by drying under vacuum.

S1. SYNTHESIS AND DOPING OF EHU-30 AND EHU-30-NH_2

S1. SYNTHESIS AND DOPING OF EHU-30 AND EHU-30-NH_2

Reagents: Zirconium(IV) propoxide [$Zr(OPr)_4$, 70% in n-propanol, Sigma-Aldrich], benzene-1,4-dicarboxylic acid [BDC: $C_8H_6O_4$, 98%, Sigma-Aldrich], 2-aminobenzene-1,4-dicarboxylic acid, [NH_2BDC: $C_8H_7NO_4$, 99%, Sigma-Aldrich], methacrylic acid [$C_4H_6O_2$, 99%, Sigma-Aldrich], methanol [MeOH, 99.9%, Sigma-Aldrich], nitric acid [HNO_3, 69%, Sigma Aldrich], hydrochloric acid [HCl, 37%, Sigma-Aldrich], N,N-dimethylformamide (DMF) [C_3H_7NO, 99.9%, Labkem], copper(II) nitrate hemi(pentahydrate) [$Cu(NO_3)_2\cdot 2.5H_2O$, 98%, Sigma-Aldrich], manganese(II) nitrate tetrahydrate [$Mn(NO_3)_2\cdot 4H_2O$, 97%, Panreac], iron(II) chloride [$FeCl_2$, 98%, Sigma-Aldrich], cobalt(II) nitrate hexahydrate [$Co(NO_3)_2\cdot 6H_2O$, 98%, Sigma-Aldrich], nickel(II) nitrate hexahydrate [$Ni(NO_3)_2\cdot 6H_2O$, 98,5%, Sigma-Aldrich] and zinc nitrate hexahydrate [$Zn(NO_3)_2\cdot 6H_2O$, 99%, Fluka].

S1.1. SYNTHESIS OF EHU-30 AND EHU-30-NH_2

EHU-30 and EHU-30-NH_2 were prepared following procedures similar to previously reported ones.[1,2] See details below. Chemical characterization of the samples was completed by PXRD, FTIR, TGA, H-NMR and ICP-OES (see below).

Synthesis of EHU-30: $Zr(OPr)_4$ (1.050 g, 2.25 mmol) and benzene-1,4-dicarboxylic acid (0.4 g, 2.4 mmol) were added into a sealed glass reactor. The mixture was stirred for 5 minutes, and then methacrylic acid was added (700 μL). The mixture was stirred for 10 minutes. Lastly, distilled water (10 μL) was added and the doughy mixture was stirred for 30 minutes. Thereafter, the flask was sealed again and the mixture was heated to 140 °C for 2 hours under continuous stirring. To the obtained product, MeOH (20 mL) was added and the mixture was stirred for 2 hours. The obtained product was washed three times with methanol, filtered under vacuum and dried at 130 °C. The resulting compound presented the aspect of a finely divided white powder.

Synthesis of EHU-30-NH_2: Zirconium(IV) propoxide (1.053 g, 2.25 mmol), isobutyric acid (1400 μL, 15 mmol) and 2-aminobenzene-1,4-dicarboxylic acid (0.412 g, 2.25 mmol) were mixed under continuous stirring in a teflon vessel. Thereafter, 10 μL (0.56 mmol) of water were added. The closed teflon vessel was placed in a preheated oven at 140°C for 4 hours. Again, the synthesis product was washed three times with methanol, filtered under vacuum and dried at 130 °C. The resulting compound appeared as yellowish fine powder.

The analysis of the linker vacancies have been performed according to previous procedures used for zirconium MOFs.[2,3] Data of the elemental analysis, NMR spectra (section S4) and thermogravimetric analysis (section S5) have been employed in order to provide a more accurate formula. To set the linker vacancies, OH^-/H_2O pairs and monocarboxylates (modulator) have been considered as the two possibilities of defect compensating ligands. Accordingly, the molecular weights obtained from the thermogravimetric analysis and the modulator/benzene-1-4,dicarboxylato ratios obtained from ^{1}H-NMR spectra have allowed to set the following formulas:

EHU-30: $[Zr_6O_4(OH)_4(C_8H_4O_4)_{4.8}(C_4H_5O_2)_{0.96}(H_2O)_{1.44}(OH)_{1.44}]$ (MW: 1599.23 g/mol).

EHU-30-NH_2: $[Zr_6O_4(OH)_4(C_8H_5NO_4)_{5.39}(C_4H_7O_2)_{0.97}(H_2O)_{0.25}(OH)_{0.25}]$ (MW: 1738.12 g/mol).

The ideal MOF formula is depicted $[Zr_6(\mu_3\text{-}O)_4(\mu_3\text{-}OH)_4(\mu_4\text{-}BDC)_6]_n$. In EHU-30 and EHU-30-$NH_2$ 1.2 and 0.61 BDC linkers per formula have been replaced by modulator molecules and H_2O/OH^-. This implies that each MOF exhibits a 20 and a 10% of linker vacancies, respectively.

S1.2. SYNTHESIS OF Cu@EHU-30 AND Cu@EHU-30-NH_2

Synthesis of Cu@EHU-30 and Cu@EHU-30-NH_2: MeOH (7 mL) was added to the copper(II) nitrate (0.017g, 0.072 mmol), and it was stirred until it became a clear solution. Then, EHU-30 (0.12 g, 0.072 mmol) was added and the suspension was stirred at room temperature for 2 hours. Consecutively, the powdered sample was collected by vacuum filtration and washed with MeOH (7 mL) for 10 minutes to remove unreacted copper(II)

salt. Finally, the obtained precipitate was collected again by vacuum filtration and dried at 80 °C for 1 h. For comparative purposes the doping process was also performed for other metal transition metal ions following the same procedure but using instead copper(II) nitrate, manganese(II) nitrate, iron(II) chloride, cobalt(II) nitrate, nickel(II) nitrate hexahydrate or zinc nitrate hexahydrate.

The chemical balance depicted in Figure 2 of the manuscript was performed analyzing by ICP-OES zirconium and copper content of both the polycrystalline product and all the filtered solution comprised by reaction solvent and the washing solvent. In this regard, the measurements of digested samples showed Cu/Zr ratios of 0.04 and 0.05 which, according to a transmetalation mechanism, it would imply to achieve cluster ratios of $\{Zr_{5.77}Cu_{0.23}\}$ and $\{Zr_{5.70}Cu_{0.30}\}$ for EHU30 and EHU-30-NH_2, respectively.

The chemical analysis performed on EHU-30 samples doped with different metal ions reveals that the doping is more efficient for copper(II), since it allows to include a noticeably greater amount of this metal into the structure. For comparative purposes Figure S1.1 shows the M/Zr ratios (M(II): Mn, Fe, Co, Ni, Cu and Zn) for each doped sample.

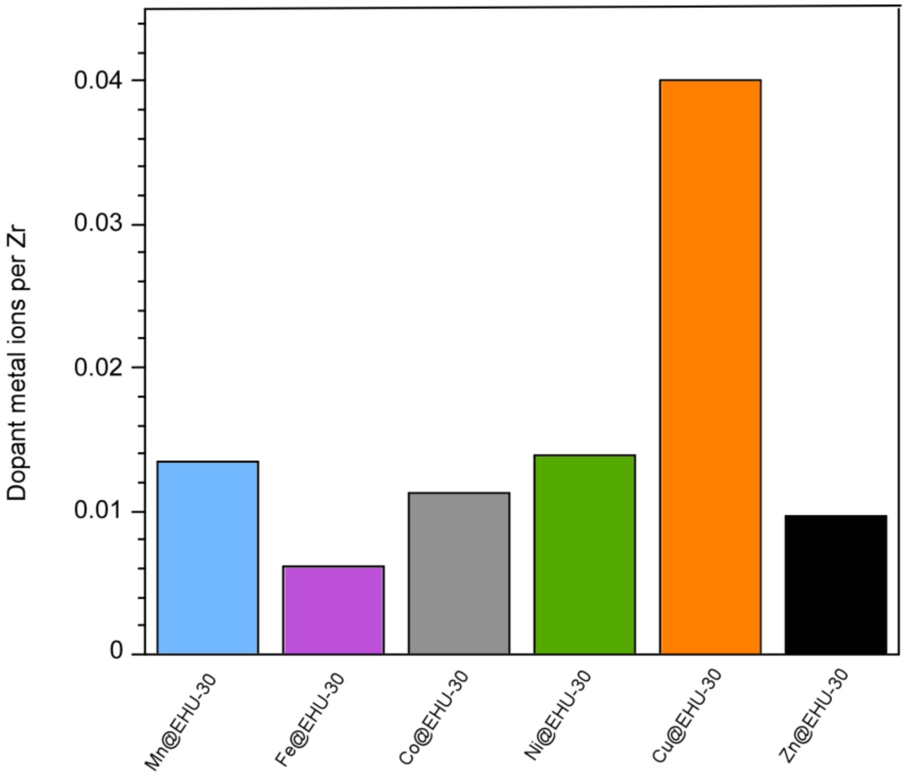

Figure S1.1 Amount of divalent transition metal ions included during the doping of EHU-30, referred as M/Zr ratio (M(II): Mn, Fe, Co, Ni, Cu, and Zn).

S2. POWDER X-RAY DIFFRACTION (PXRD) ANALYSIS

S2. POWDER X-RAY DIFFRACTION (PXRD) ANALYSIS

The crystallinity and purity of the samples have been assessed by powder X-ray diffraction. Figure S2.1 gathers the experimental PXRD patterns of parent and doped MOFs in comparison with the simulated ones. The pattern-matching analysis of all the samples (Figure S2.2), performed using the space group and cell parameters of EHU-30[¡Error! Marcador no definido.] and EHU-30-NH_2,[2] shows a good agreement as it can be inferred from the similar cell parameters and low disagreement factors (Table S2.1-Table S2.4). According to the profile fitting performed on the PXRD data, the Cu-doping does not exert any meaningful change in the crystal structure parameters which consistent with robustness of the Zr-MOF.

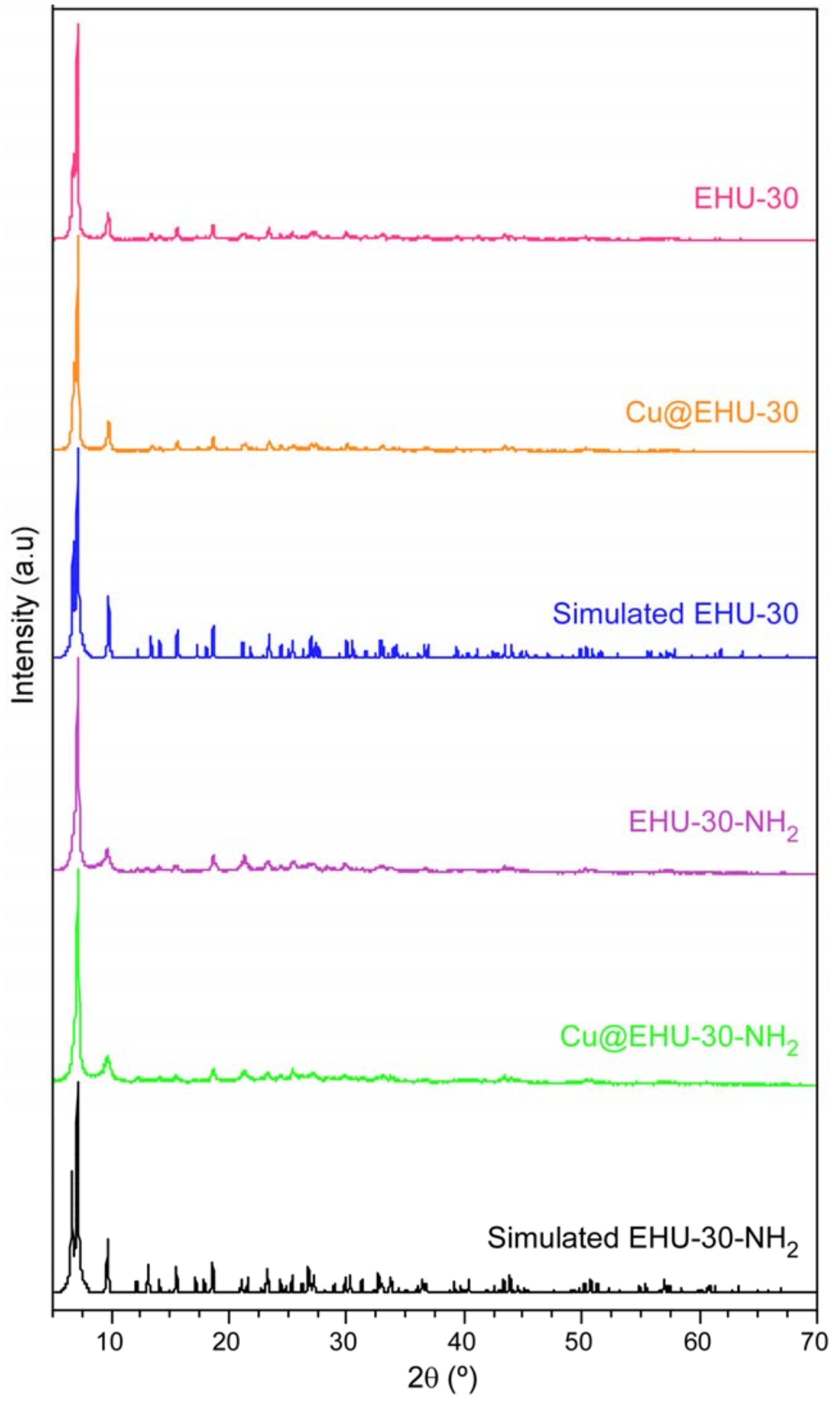

Figure S2.1 PXRD patterns for parent and Cu(II)-doped metal-organic frameworks in comparison with simulated diffractograms of EHU-30 and EHU-30-NH_2.

Table S2.1 Cell parameters and factors obtained by pattern-matching analysis made on EHU-30.

**Reported for EHU-30**		**Experimental profile fitting**	
Space group	*P*6$_3$*/mmc*	Space group	*P*6$_3$*/mmc*
a (Å)	14.6690(4)	a (Å)	14.666(3)
c (Å)	26.8478(1)	c (Å)	26.883(8)
V (Å^3)	5003.10	V (Å^3)	5008.07
		R_p	11.3
		R_{wp}	14.4
		R_{exp}	7.72
		χ^2	3.48

$R_p=100\cdot(\sum_{i=l,n}|y_i - y_{c,i}|)/(\sum_{i=l,n} yi)$; $R_{wp}= \surd[(\sum w_i(y_i(obs)-y_i(calc)^2)/ (\sum w_i y_i^2(obs))]$; $R_{exp}= \surd[(N-P-C)/ \sum w_i y_i^2(obs)]$ and $\chi^2= [R_{wp}/R_{exp}]^2$.

Table S2.2 Cell parameters and factors obtained by pattern-matching analysis made on EHU-30-NH_2

**Reported for EHU-30-NH_2**		**Experimental profile fitting**	
Space group	*P*6$_3$*/mmc*	Space group	*P*6$_3$*/mmc*
a (Å)	14.7044(12)	a (Å)	14.6741(4)
c (Å)	27.320(7)	c (Å)	27.363(1)
V (Å^3)	5115.71	V (Å^3)	5102.77
		R_p	9.60
		R_{wp}	13.8
		R_{exp}	7.10
		χ^2	3.79

$R_p=100\cdot(\sum_{i=l,n}|y_i - y_{c,i}|)/(\sum_{i=l,n} yi)$; $R_{wp}= \surd[(\sum w_i(y_i(obs)-y_i(calc)^2)/ (\sum w_i y_i^2(obs))]$; $R_{exp}= \surd[(N-P-C)/ \sum w_i y_i^2(obs)]$ and $\chi^2= [R_{wp}/R_{exp}]^2$.

Table S2.3 Cell parameters and factors obtained by pattern-matching analysis made on Cu@EHU-30.

**Reported for EHU-30**		**Experimental profile fitting**	
Space group	*P*6$_3$*/mmc*	Space group	*P*6$_3$*/mmc*
a (Å)	14.6690(4)	a (Å)	14.6630(2)
c (Å)	26.8478(1)	c (Å)	26.6305(6)
V (Å^3)	5003.10	V (Å^3)	4958.59
		R_p	9.28
		R_{wp}	12.9
		R_{exp}	6.97
		χ^2	3.41

$R_p=100\cdot(\sum_{i=l,n}|y_i - y_{c,i}|)/(\sum_{i=l,n} yi)$; $R_{wp}= \surd[(\sum w_i(y_i(obs)-y_i(calc)^2)/ (\sum w_i y_i^2(obs))]$; $R_{exp}= \surd[(N-P-C)/ \sum w_i y_i^2(obs)]$ and $\chi^2= [R_{wp}/R_{exp}]^2$.

Table S2.4 Cell parameters and factors obtained by pattern-matching analysis made on Cu@EHU-30-NH_2.

**Reported for EHU-30-NH_2**		**Experimental profile fitting**	
Space group	*P*6$_3$*/mmc*	Space group	*P*6$_3$*/mmc*
a (Å)	14.7044(12)	a (Å)	14.6955(3)
c (Å)	27.320(7)	c (Å)	27.305(1)
V (Å^3)	5115.71	V (Å^3)	5106.84
		R_p	8.87
		R_{wp}	11.9
		R_{exp}	7.52
		χ^2	2.52

S3. FOURIER-TRANSFORM INFRARED SPECTRA (FTIR)

S3. FOURIER-TRANSFORM INFRARED SPRECTA (FTIR)

Figure S3.1 shows a comparison of the infrared spectra of EHU-30, EHU-30-NH_2, Cu@EHU-30 and Cu@EHU-30-NH_2 samples. The assignation of the main vibration modes are gathered Table S3.1. The large O–H peaks in the IR spectra are indicative of H_2O/OH^- pairs that replace the missing linkers in the structure (see below further details in thermogravimetric and NMR analysis).

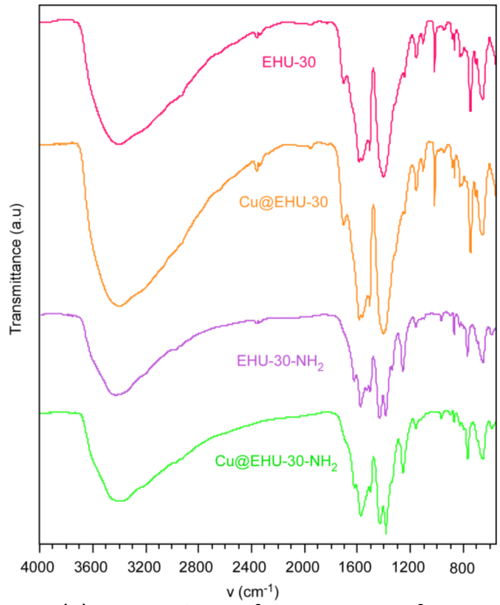

Figure S3.1 (a) Comparison of FTIR spectra for parent and doped MOFs.

Table S3.1 Band assignments FTIR spectra for parent and doped MOFs.[a]

$\bar{\nu}$ (cm^{-1})	EHU-30<br>Cu@EHU-30	EHU-30-NH_2<br>Cu@EHU-30-NH_2
ν (OH)	3000-3700, m	3000-3700, m
ν_{asym} (NH_2)	--	3400-3500, m
ν (C–H)	3000-2850, w-m	3000 - 2850, w-m
ν_{asym} (COO)	1700 m	1700 m
ν (C_{ar}–C)	1575, s	1575, s
ν_{ring} (C_{ar}–C_{ar})	1500, m	1500, m
ν_S (COO)	1425, s	1430, s
ν (Car-N)	--	1380, s
δ_{ip} (NH_2)	--	1336, sh
δ_{oop} (NH_2)	--	1255, m
δ_{oop} (C-H)	750, s	770, s
ν (M–Ooxide)	650, m	650, m
ν (M–Ocoo)	550, m	580, m
ν (M–O_{OH})	490, m	490, m

[a]: s: strong, m: medium, w: weak and sh: shoulder signals; ν: stretching, δ: bending, sym: symmetric, asym: antisymmetric, ip: in plane, ring: ring stretching, ar: aromatic, oop: out of plane.

S4. NUCLEAR MAGNETIC RESONANCE (NMR) SPECTROSCOPY

S4. NUCLEAR MAGNETIC RESONANCE (NMR) SPECTROSCOPY

Prior to measure the ^{1}H-NMR spectra (500 MHz), 70 mg of each sample (EHU-30 and EHU-30-NH_2) were digested in 2 mL of a 1 M NaOH solution (in deuterated water, D_2O) during 24 h. Thereafter the solid residue corresponding to ZrO_2 was filtered off. The NMR spectra were taken on the liquid fraction. Figures S4.1 and S4.2 show the peak integration and the label assignation of the organic species present in digested EHU-30 and EHU-30-NH_2 samples. The residual signal of the solvent (D_2O) appears at 4.79 ppm.

EHU-30: The singlet at 7.72 ppm is due to the aromatic signals of benzene-1,4-dicarboxylic acid. Around 5.0 ppm, the two characteristic singlets of the olefinic protons of methacrylic acid (modulator) are observed, at 5.18 and 5.5 ppm. The singlet 3.24 ppm is related to methanol hosted in the pores of the MOF. Likewise, the signal of the methyl group of methacrylic acid (which integrates 3:1 considering the methylene group) can be found at 1.71 ppm. The integration of those signals indicates that the amount of methacrylic acid after the digestion of EHU-30 is 17% relative to all carboxylic ligands (modulator plus benzene-1,4-dicarboxylic acid). This result fits properly with the values estimated from the thermogravimetric analysis that will be later discussed.

EHU-30-NH$_2$: The three signals with different multiplicity around 7 ppm are attributed to the aromatic protons of 2-aminobenzene-1,4-dicarboxylic acid. Again methanol hosted in the pores of the compound leads to a singlet at 3.23 ppm. Two additional signals (a multiplet at 2 ppm that integrates to one proton and a doublet at 1 ppm that integrates to six protons) are related to the presence of a small amount of isobutyric acid (modulator). The integration of the signals indicates that the amount of isobutyric acid is 15% relative to all carboxylic ligands (modulator plus 2-aminobenzene-1,4-dicarboxylic acid). Again this value fits fairly well to that estimated from the TGA analysis.

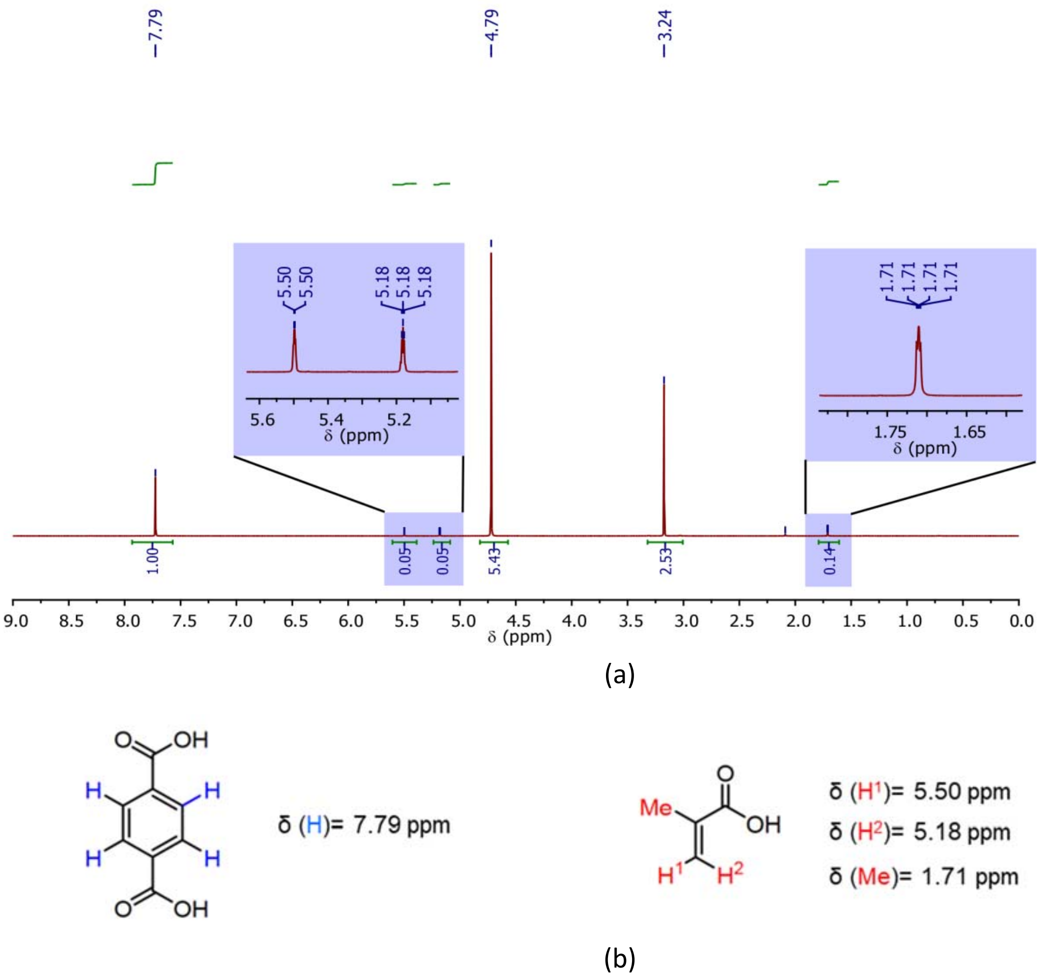

Figure S4.1 (a) ^{1}H-NMR spectrum of digested EHU-30. (b) Label assignation of organic compounds: benzene-1,4-dicarboxylic acid in blue and methacrylic acid in red.

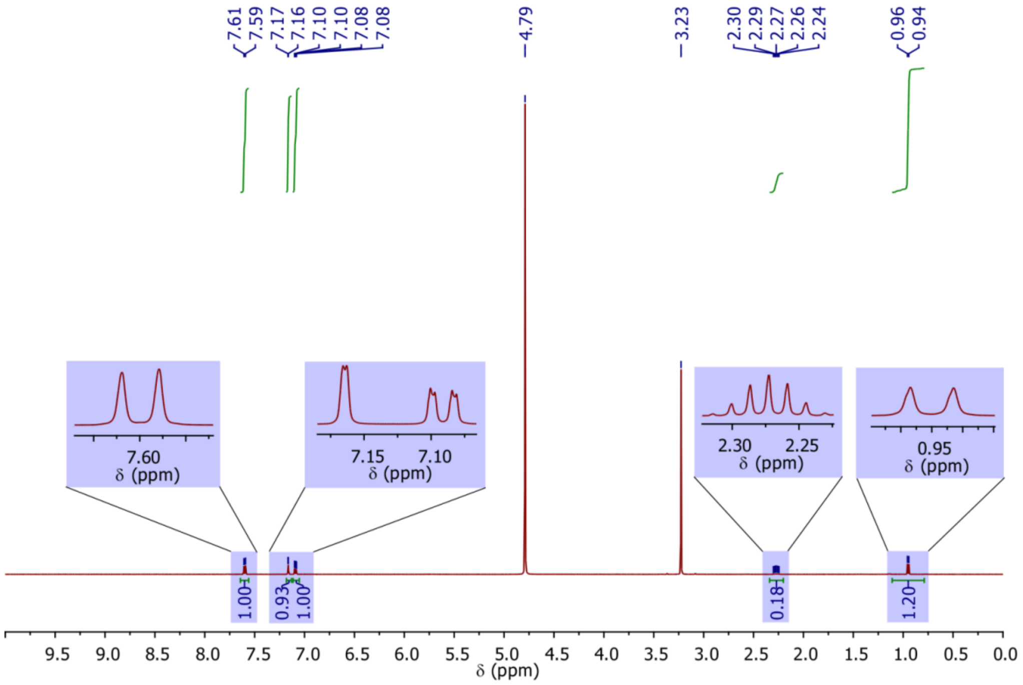

(a)

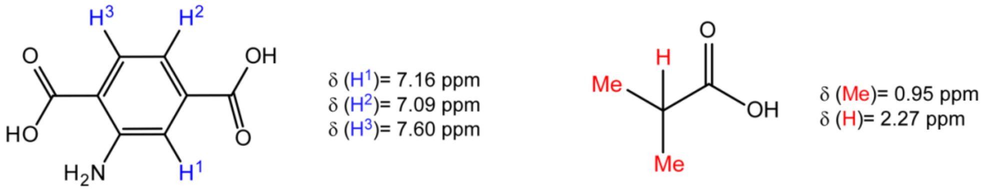

(b)

Figure S4.2 (a) ^{1}H-NMR spectrum of digested EHU-30-NH_2. (b) Label assignation of organic compounds: 2-aminobenzene-1,4-dicarboxylic acid in blue and isobutyric acid in red.

S5. THERMOGRAVIMETRIC ANALYSIS

S5. THERMOGRAVIMETRIC ANALYSIS

Figures S5.1 shows the result of the thermogravimetric analysis of EHU-30 and EHU-30-NH_2. Thermogravimetric curves exhibit an analogous behavior to that of other zirconium MOFs. First, at 25–110 °C range, moisture or solvent molecules (MeOH) hosted in pores are lost. Then, at 110–400 °C range, there is a less pronounced drop in weight that can be ascribed to a series of successive processes. Firstly, it can be attributed to the loss of coordinating water molecules. Thereafter, water molecules formed from the condensation of hydroxide groups. Finally, at 350–600 °C range, the framework decomposition takes place, which leads to a very intense exothermic peak. This leaves ZrO_2 as a final residue, which has been identified by PXRD.

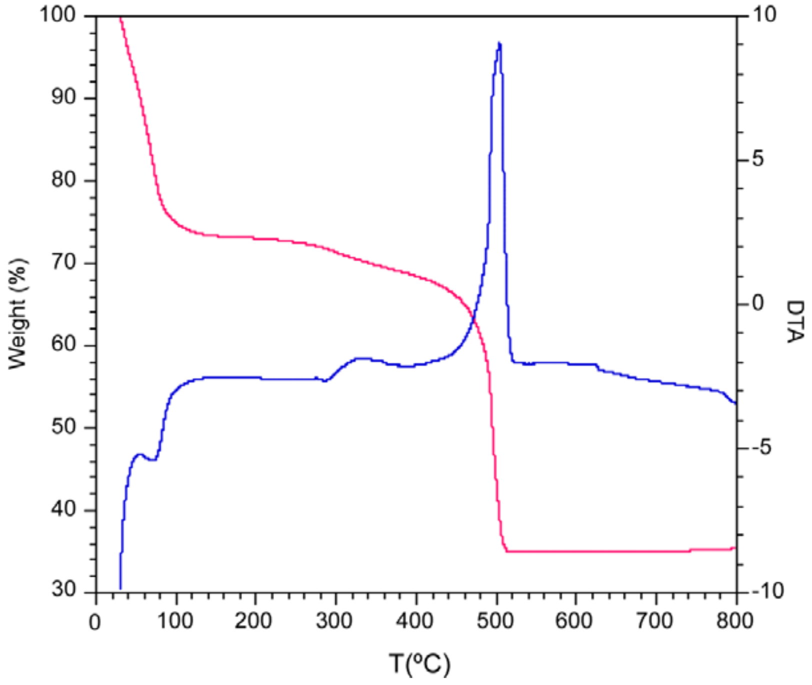

(a)

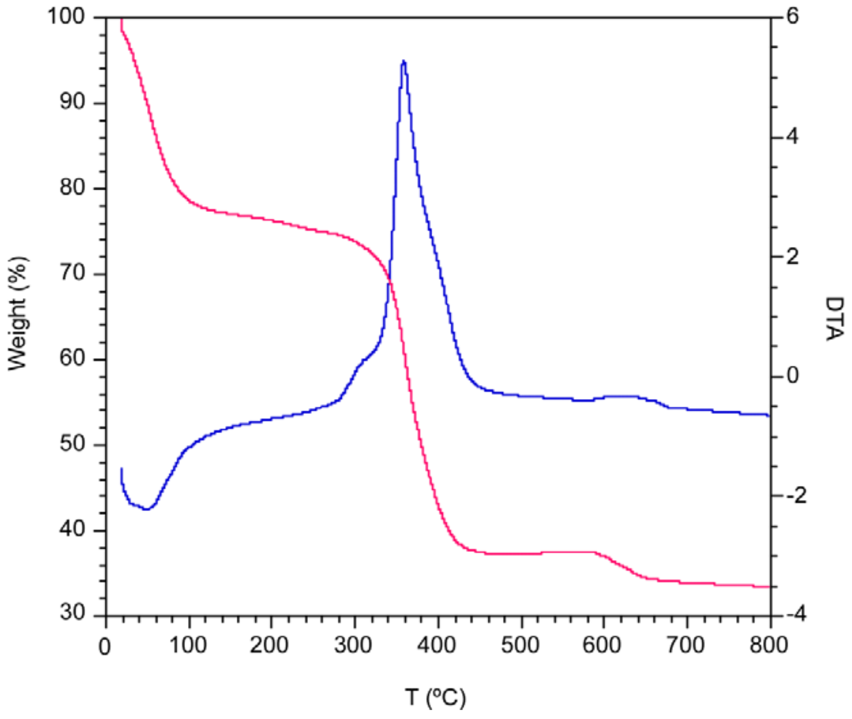

(a)

Figure S5.1 TGA-DTA curves of (a) EHU-30 and (b) EHU-30-NH_2.

S6. SYNCHROTRON TOTAL SCATTERING DATA AND PAIR DISTRIBUTION FUNCTION ANALYSIS

S6. SYNCHROTRON RADIATION AND PAIR DISTRIBUTION FUNCTION ANALYSIS

Powder samples were packed in glass capillaries with a diameter of 0.8 mm. Total X-ray scattering data suitable for PDF analyses were collected at the P02.1 beamline at PETRA III, DESY (Hamburg, Germany), using monochromatic synchrotron X-rays with a fixed energy of 60.0 keV (0.2068 Å). The patterns were collected with a Varex XRD 4343CT flat panel detector (150 × 150 μm^2 pixel size, 2880 x 2880 pixel area) with a sample-to-detector distance (SDD) of 292 mm and exposure times of 1800 seconds, capturing quarters of the Debye-Scherrer rings. Calibration of the detector and the SDD was performed measuring LaB_6 (NIST 660b) as standard material. An empty capillary was measured in order to compensate for the contribution of glass to the diffraction patterns. PDFs were obtained from the total scattering data within xPDFsuite[4] to a Qmax = 22 $Å^{-1}$. Differential PDFs were calculated by subtracting the PDF of the pristine EHU-30 from that of the copper metalated material. Simulated PDF data of the EHU-30 framework was obtained using the PDFgui software.[5]

S7. GAS ADSORPTION DATA

S7. GAS ADSORPTION DATA

BET fitting experimental isotherms

N_2 adsorption isotherms (77 K) are gathered in Figure S7.1. All of them resemble a type I isotherm as expected for microporous materials. The micropore filling takes place below ca. 0.1 relative pressure. Thereafter, a monotonic increase of the adsorption followed by a marked step at $P/P_0 > 0.9$ is observed, which can be related to the multilayer adsorption into the external surface of the particles (nanocrystallites) and to the interparticle condensation phenomena, respectively. The high relative pressure at which the latter step takes place and the absence of a hysteresis cycle indicates that interparticle pores are mainly macroporous (> 50 nm).

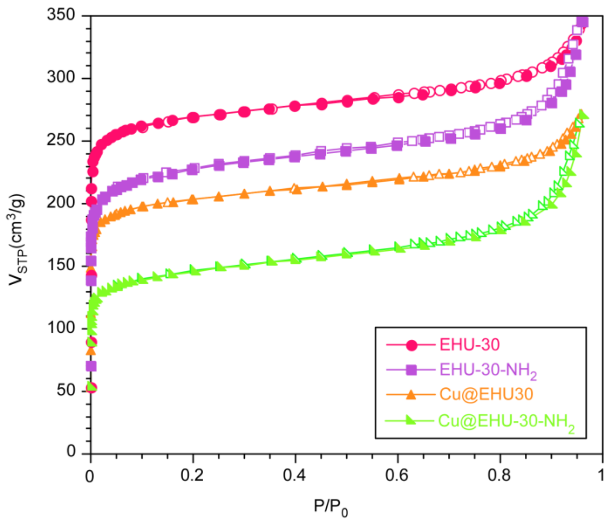

Figure S7.1 Nitrogen adsorption isotherm for EHU-30, Cu@EHU-30, EHU-30-NH_2 and Cu@EHU-30-NH_2.

Surface area values were calculated using Brunauer-Emmett-Teller[6] equation S7.1.

$$\frac{1}{v\left[(p_0/p)-1\right]} = \frac{c-1}{v_m c}\left(\frac{p}{p_0}\right) + \frac{1}{v_m c} \qquad \text{(S7.1)}$$

V is the specific amount of N_2 adsorbed at relative pressure P/P_0, V_m is the specific amount of N_2 adsorbed corresponding to the monolayer formation and C is a parameter which is exponentially related to the energy of monolayer formation.

In order to avoid ambiguities when describing the surface area of microporous MOFs,[7] the pressure range for the data fitting was fixed according to the consistency criteria proposed by Roquerol et al.:[8] the pressure range selected should have values of $V(1 - P/P_0)$ increasing with P/P_0, the points used to calculate the BET surface area must be linear with an upward slope in such a way that the linear regression must yield a positive y-intercept (a positive C value) and the P/P_0 value corresponding to V_m should be within the BET fitting range.

As stated by the first consistency criteria, $V(1 - P/P_0)$ vs P/P_0 plots were used to define the pressure range (Figure S7.2). Second and third criteria were as well fulfilled upon the selected fitting range. BET fitting parameters and results for EHU-30 and doped samples are shown in Table S7.1.

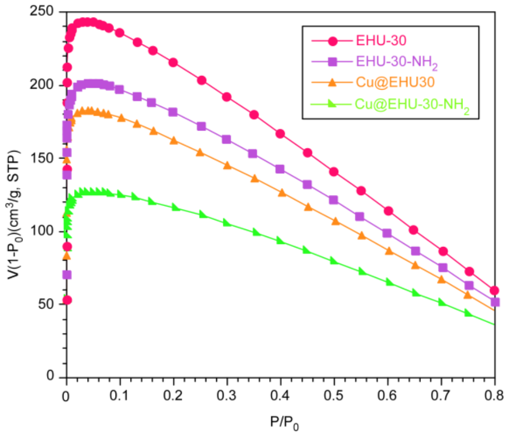

Figure S7.2. Consistency plot [V(1 – P/P₀) vs. P/P₀] upon N_2 adsorption isotherms.

Table S7.1 BET fitting details for experimental N_2 isotherms.

Code	P/P_0 range	S_{BET} (m^2/g)	R^2	C	V_m (cm^3/g)	P/P_0 for V_m (interpolation)
**EHU-30**	0.0009–0.0304	1065	0.99999	5838.8	233.10	0.01368
**Cu@EHU-30**	0.00201–0.0397	801	0.99999	3242.2	183.95	0.01831
**EHU-30-NH$_2$**	0.0006–0.0206	885	0.99997	2489.2	203.11	0.01053
**Cu@EHU-30-NH$_2$**	0.0041–0.0048	558	0.99999	2281.3	128.13	0.02113

CO_2 adsorption isotherms and calculation of isosteric heats of adsorption

CO_2 adsorption isotherms were measured at 273 and 298 K for EHU-30, Cu@EHU-30, EHU-30-NH$_2$ and Cu@EHU30-NH$_2$ (Figure S7.3). The lowering of CO_2 uptake capacity at medium-high pressures follows the same trend to that observed for N_2 isotherms

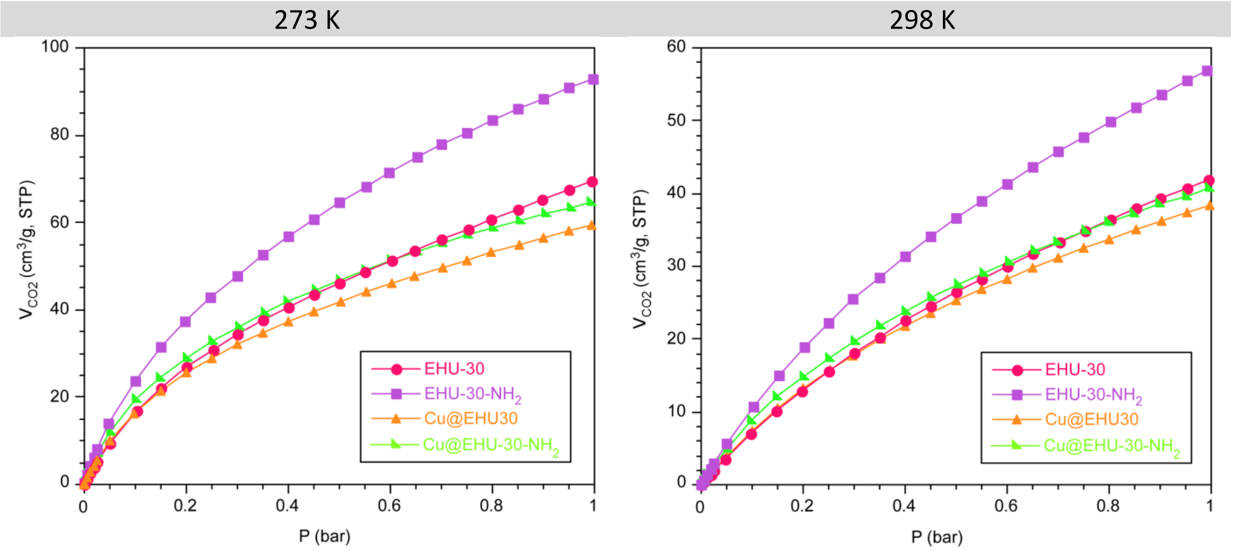

Figure S7.3 CO_2 adsorption isotherms at 273 and 298 K for EHU-30, Cu@EHU-30, EHU-30-NH$_2$ and Cu@EHU30-NH$_2$.

With the aim of estimating CO_2 adsorption enthalpies, the isotherms were fitted to modified Clausius-Clapeyron equation.[9] Figure S7.3 shows the calculated isosteric heats of adsorption (Q_{st}). In all cases the isosteric heats fall progressively with the increase of the gas uptake. This trend is reasonable as CO_2 molecules bind to most energetically favourable positions and once these positions are occupied, the rest of the positions are filled, which as a result leads to a progressive drop in isosteric heats as the occupancy of the pore increases. Interestingly, at near zero loading, Cu@EHU-30 and Cu@EHU-30-NH_2 exhibit a somewhat greater isosteric heat value than parent EHU-30 and EHU-30-NH_2. Similarly, the amino-functionalized compound exhibit higher Q_{st} values than their unfunctionalized counterparts. This behaviour can be explained by a more favourable interaction of the CO_2 with the dopants and the amino group.

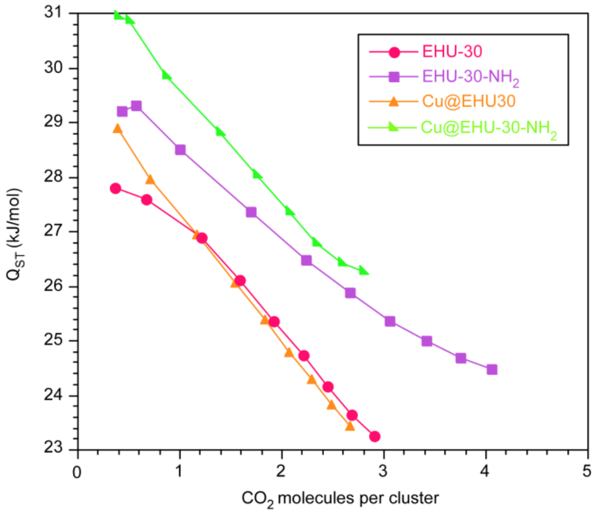

Figure S7.3 Calculated isosteric heats of adsorption for CO_2.

S8. CONTINUOUS CO_2 ELECTROREDUCTION EXPERIMENTS

S8. CONTINUOUS CO_2 ELECTROREDUCTION EXPERIMENTS

Preparation of MOF-GDEs

The procedure to prepare the GDEs (A= 10 cm^2) has been described in detail before.[10] The catalytic ink was airbrushed onto a TGP-H-60 (Toray Inc.) porous carbon with an optimum catalyst loading of 1 $mg\cdot cm^{-2}$.[11,12] The catalytic ink is formed by the MOF, Nafion® dispersion 5 wt.% (Alfa Aesar) as binder, and isopropanol (Sigma Aldrich) as vehicle, with a 70:30 catalyst/Nafion mass ratio and a 3% solids (catalyst + Nafion) percentage. The mixture is sonicated for at least 30 minutes to obtain a homogeneous slurry that is subsequently airbrushed on the surface of the porous substrate. The carbon substrate minimizes the transport resistance, contributing to an efficient transfer of CO_2 and diffusion of products.[13]

Electrochemical experiments

The prepared materials are evaluated for the continuous electroreduction of CO_2 using a filter-press electrochemical cell (Micro Flow Cell, ElectroCell A/S) at ambient conditions. A Nafion® 117 cation exchange membrane was used to separate the cathode and anode compartments. The airbrushed MOF-catalysed papers were employed as the working electrodes, together with a platinised titanium electrode as the anode and a Ag/AgCl (sat. KCl) reference electrode assembled close to the cathode as described before.[¡Error! Marcador no definido.] The cathode side of the reactor was fed with CO_2 gas (99.99%) with a flow/area ratio of Q_g/A= 20 $ml\cdot min^{-1}\cdot cm^{-2}$. A 0.5 M $KHCO_3$ (Panreac) aqueous solution is used as both, catholyte and anolyte, with a flow rate of Q_e/A= 2 $ml\cdot min^{-1}\cdot cm^{-2}$. The electrolytes were pumped from catholyte and anolyte tanks to the cell by two peristaltic pumps (Watson Marlow 320, Watson Marlow Pumps Group). Prior to the experiments, the aqueous electrolyte was saturated with CO_2 by bubbling for 30 min. In this study, the filter-press electrochemical system possesses three inputs (catholyte, anolyte and CO_2 separately) and two outputs (catholyte-CO_2 and anolyte).

All the experiments were performed at galvanostatic conditions (i.e. at a constant current density), using an AutoLab PGSTAT 302N potentiostat (Metrohm, Autolab B.V.). The current density was j= 10 $mA\cdot cm^{-2}$ in all the experiments. This current density value was selected according to the optimum performance set in a previous work using copper-based metal-organic materials.[¡Error! Marcador no definido.] The experimental time was 90 min, except for the stability test where the material was evaluated for 400 min. To quantify the concentration of each product, the samples were analysed in a headspace gas chromatograph (GCMS-QP2010, Ultra Shimadzu) equipped with a flame ionization detector (FID). Formate ($HCOO^-$) concentration was analyzed by Ion Chromatography (Dionex ICS 1100). Liquid samples were taken every 15 min from the catholyte tank. Besides, gas-phase reduction products were analyzed in a gas microchromatograph (3000 Micro GC, Inficon) equipped with thermal conductivity detector (TCD). The outlet gas stream is measured every 5 min. The concentration of every product was averaged from at least three replicates with a maximum standard deviation of 15.6 %.

The performance of the electrochemical process is evaluated by the rate of product formation, r (i.e. product obtained per unit of cathode area and time), and the Faradaic efficiency, FE (i.e. selectivity of the reaction for the formation of the different products), according to the following equation:

$$FE(\%) = \frac{z\cdot n\cdot F}{q}\cdot 100 \qquad (1)$$

where z is the theoretical number of electrons exchanged to form the desired product, n is the number of moles produced, F is the Faradaic constant (F= 96,485 $C\cdot mol^{-1}$) and q is the total charge applied in the process.

Table S8.1 FEs for the continuous electroreduction of CO_2 and H_2 evolution using EHU-30-based GDEs (j= 10 $mAcm^{-2}$; Q_e/A= 2 $mlmin^{-1}cm^{-2}$, Q_g/A= 20 $mlmin^{-1}cm^{-2}$).[a]

**Material**	***E*** **(V vs. Ag/AgCl)**	***FE (%)***						
		CO	HCOOH	*CH_3OH*	*C_2H_4*	*TOTAL (CO_2 products)*	*H_2*	*TOTAL*
EHU-30	-2.01	3.3±0.8	8.4±1.1	-	-	11.7	77.5	89.2
EHU-30-NH_2	-1.82	5.6±1.1	10.5±1.8	-	-	16.1	71.6	87.7
Cu@EHU-30	-1.54	5.1±0.4	7.9±0.8	2.9±0.7	4.1±0.5	20.1	63.6	83.7
Cu@EHU-30-NH_2	-1.46	7.6±0.6	9.6±1.4	6.1±2.1	7.5±1	30.9	51.8	82.7

[a]: Traces of ethanol were detected.

Table S8.2 Time-dependence on total FE (CO_2 products) with Cu@EHU-30-NH_2 (j= 10 $mAcm^{-2}$; Q_e/A= 2 $mlmin^{-1}cm^{-2}$, Q_g/A= 20 $mlmin^{-1}cm^{-2}$).

**Time (min)**	**E (V vs. Ag/AgCl)**	***FE_{TOTAL} (%)***
15	-1.59	21.2±2
30	-1.62	26.6±2.9
45	-1.51	28.5±1.8
60	-1.48	30.9±2.2
75	-1.46	30.9±3
90	-1.46	30.8±3.1
120	-1.48	31.2±1.9
150	-1.49	32.4±2.2
220	-1.51	30.7±1.4
250	-1.55	29.6±2
280	-1.56	29.9±1
400	-1.6	27.7±0.9

S9. POST-REACTION CHARACTERIZATION

S9. POST-REACTION CHARACTERIZATION

SEM, FTIR, and H[1]-NMR analyses were performed for the GDE containing Cu@EHU-30-NH_2 catalyst before and after the CO_2 reduction reaction. SEM images (Figure S9.1) show microcrystallites of Cu@EHU-30-NH_2 dispersed in Nafion. Note that not all the material can be observed as part is masked by the Nafion. The comparison of the images suggests that some material is leached during the reaction. The H[1]-NMR spectra (Figure S9.2) show that the signals corresponding to the aromatic ring of NH_2BDC ligand remain invariable, ensuring the stability of the aromatic ligand, including the amino group, during the reaction. The FTIR spectra collected by attenuated total reflectance (ATR) mode (Figure S9.3) show that the main vibration modes of the metal-organic framework persist (Table S3.1) indicating that the overall molecular structure is retained. The differences between the FTIR spectra can be related with the presence of adsorbed electrolyte and reaction products. Furthermore, both spectra show the symmetric and antisymmetric stretching bands of CF_2 groups of Nafion around 1210 and 1145 cm^{-1}, respectively.

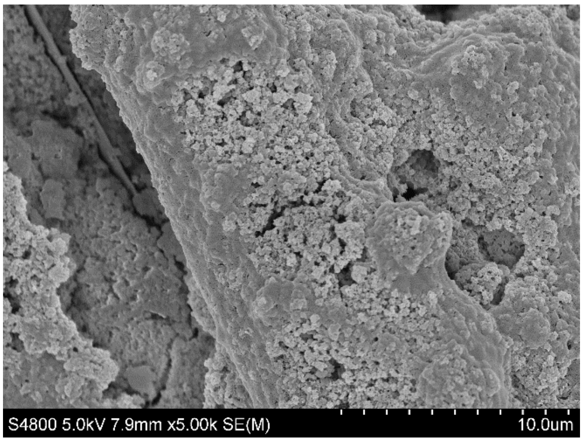

(a)

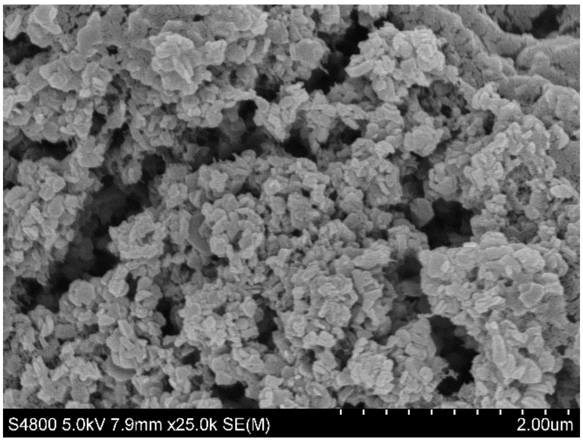

(b)

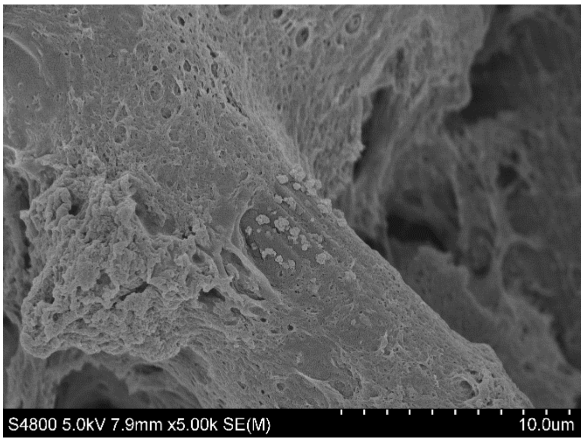

(c)

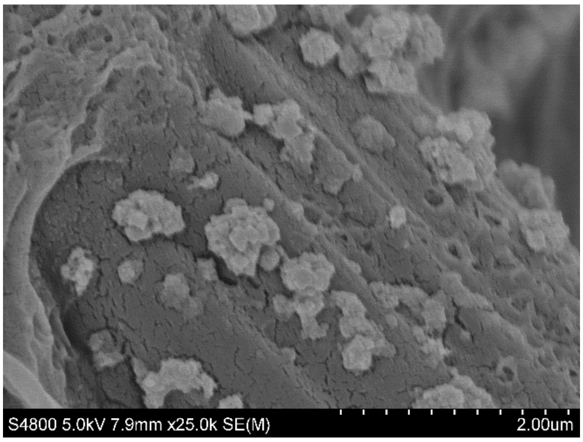

(d)

Figure S9.1 SEM images taken on GDEs containing Cu@EHU-30-NH_2 catalyst (a, b) before and (c, d) after the CO_2 reduction reaction.

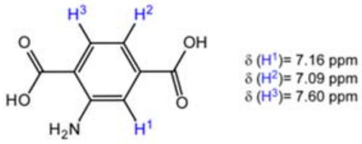

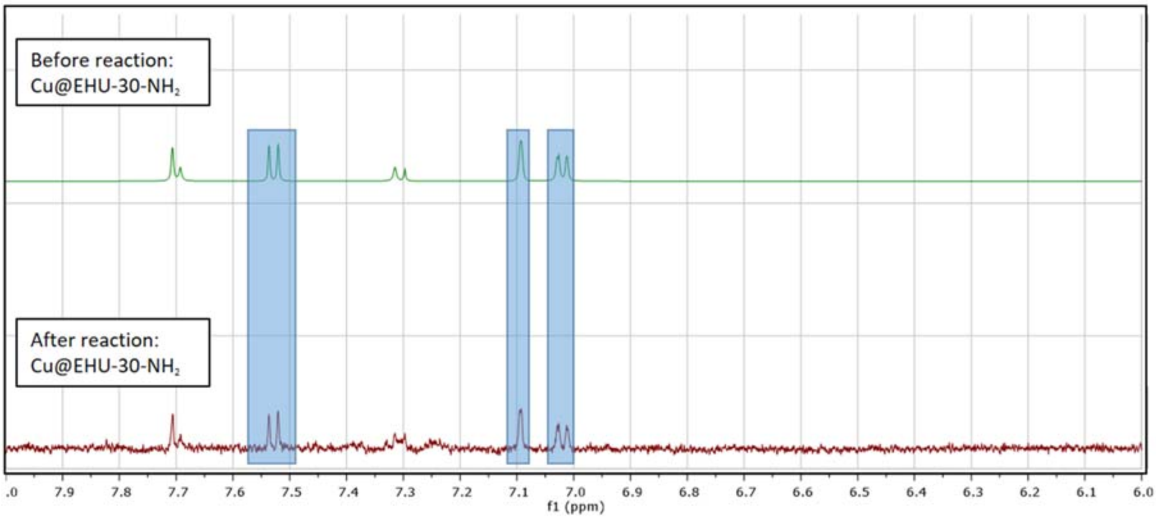

Figure S9.2 H[1]-NMR spectra collected for Cu@EHU-30-NH$_2$ before and after the CO_2 reduction reaction**.**

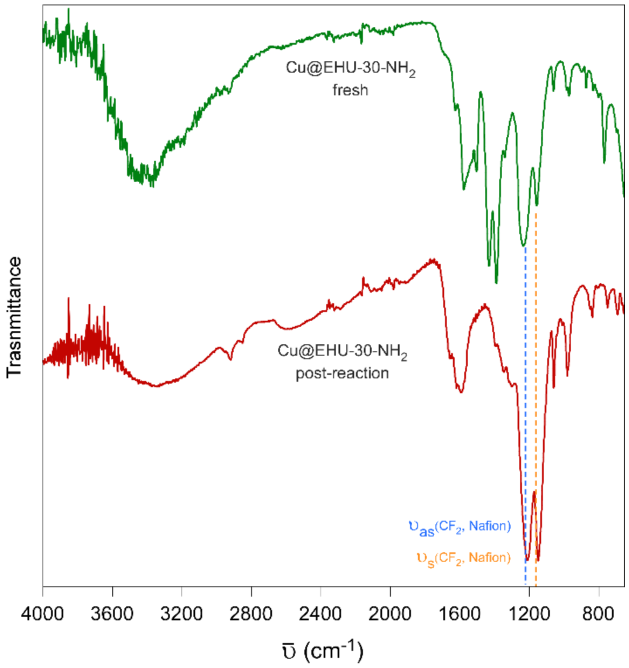

Figure S9.3 FTIR-ATR spectra taken on GDEs containing Cu@EHU-30-NH$_2$ catalyst before (green, up) and after (red, down) the CO_2 reduction reaction.

S10. REFERENCES

S10. REFERENCES

[1] Perfecto-Irigaray, M. *et al.* [$Zr_6O_4(OH)_4$(benzene-1,4-dicarboxylato)$_6$]$_n$: A hexagonal polymorph of UiO-66. *Chem. Commun.* **55**, 5954–5957 (2019).

[2] Perfecto-Irigaray, M. *et al.* Metastable Zr/Hf-MOFs: The hexagonal family of EHU-30 and their water-sorption induced structural transformation. *Inorg. Chem. Front.* **8**, 4767–4779 (2021).

[3] Taddei, M, *et al.* Band gap modulation in zirconium-based metal–organic frameworks by defect engineering. *J. Mater. Chem. A* **7**, 23781-23786 (2019).

[4] Yang, X., Juhas, P., Farrow, C. L. & Billinge, S. J. L. XPDFsuite: An end-to-end software solution for high throughput pair distribution function transformation, visualization and analysis. arXiv 2014, 1402.3163. arXiv.org e-Print archive. https://arxiv.org/abs/1402.3163v3 (accessed Sept 1, 2021).

[5] Farrow, C. L *et al.* PDFfit2 and PDFgui: computer programs for studying nanostructure in crystals. *J. Phys. Condens. Matter.* **19**, 335219 (2007).

[6] Brunauer, S., Emmett P.H. & Teller E. J. Adsorption of gases in multimolecular layers. *Am. Chem. Soc.* **60**, 309–319 (1938).

[7] Thommes, M. *et al.* Physisorption of gases, with special reference to the evaluation of surface area and pore size distribution (IUPAC Technical Report). *Pure Appl. Chem.* **87**, 1051–1069 (2015).

[8] Rouquerol, J., Llewellyn, P. & Rouquerol, F. Is the bet equation applicable to microporous adsorbents?. *Stud. Surf. Sci. Catal.*, **160**, 49–56 (2007).

[9] (a) Sumida, K. *et al.* Carbon dioxide capture in metal–organic frameworks, *Chem. Rev.* **112**, 724–781 (2012). (b) Pan, H., Ritter, J. A. & Balbuena, P. B. Examination of the approximations used in determining the isosteric heat of adsorption from the Clausius−Clapeyron equation. *Langmuir* **14**, 6323–6327 (1998).

[10] Albo J. *et al*. Copper-based metal–organic porous materials for CO_2 electrocatalytic reduction to alcohols. *ChemSusChem*. **10**, 1100-1109 (2017).

[11] Albo J., Perfecto-Irigaray M., Beobide G. & Irabien A. Cu/Bi metal-organic framework-based systems for an enhanced electrochemical transformation of CO2 to alcohols. *J. CO2 Util.* **33**, 157–165 (2019).

[12] Albo J., Sáez A., Solla-Gullón J., Montiel V. & Irabien A. Production of methanol from CO_2 electroreduction at Cu_2O and Cu_2O/ZnO-based electrodes in aqueous solution, *Appl. Catal. B-Environ*. **176**, 709-717 (2015).

[13] Albo J. & Irabien A. Cu_2O-loaded gas diffusion electrodes for the continuous electrochemical reduction of CO_2 to methanol. *J. Catal.* **343**, 232-239 (2016).
